# Prevalence and predictors of clinic appointment non-adherence among adults with poorly controlled hypertension in a primary care setting

**DOI:** 10.4314/gmj.v55i4.4

**Published:** 2021-12

**Authors:** Godpower C Michael, Salihu T Tanimu, Ibrahim Aliyu, Bukar A Grema, Haliru Ibrahim, Abubakar A Mohammed, Yahkub B Mutalub

**Affiliations:** 1 Department of Family Medicine, Aminu Kano Teaching Hospital, Kano, Nigeria; 2 Department of Paediatrics, Bayero University Kano/Aminu Kano Teaching Hospital, Kano, Nigeria; 3 Department of Medicine, Federal Medical Centre, Birnin Kudu, Nigeria; 4 Department of Family Medicine, University of Maiduguri Teaching Hospital, Maiduguri, Nigeria; 5 Department of Family Medicine, Abubakar Tafawa Balewa University Teaching Hospital, Bauchi, Nigeria

**Keywords:** Hypertension, missed appointment, clinic appointment non-adherence, predictors, primary care

## Abstract

**Objectives:**

To assess the prevalence and predictors of non-adherence to clinic appointments in adult patients with poorly controlled hypertension.

**Design:**

A descriptive cross-sectional study.

**Setting:**

A primary care setting (family medicine clinic) overseen by family physicians in Kano, Nigeria.

**Participants:**

Two hundred and thirty-four randomly selected patients, aged ≥ 18 years with a diagnosis of hypertension, who had been on treatment for ≥1 year and had a current blood pressure of ≥140/90 mmHg were included.

**Main outcome measures:**

Non-adherence to clinic appointment among participants

**Results:**

Participants' mean age was 55±12.2 years (range: 23–85 years); they were predominantly females (163, 69.7%). Sixty (25.6%) participants were non-adherent to clinic-appointments. Being employed (OR [Odds ratio] =2.92, 95%CI [confident interval] =1.52–5.65, *P*=0.002), inability of participants or their children to pay the medical bills (OR=2.92,95%CI=1.42–6.00, *P*=0.004), and systolic blood pressure (SBP) of <160mmHg (OR=0.43, 95%CI=0.22–0.86, *P*=0.018) were predictors of clinic appointment non-adherence.

**Conclusions:**

The prevalence of non-adherence to clinic appointments was high. Being employed, patients or their children's inability to pay medical bills, and higher SBP were predictors of non-adherence to clinic appointments. Therefore, more studies are needed on effective interventions to reduce non-adherence to clinic appointments in this setting.

## Introduction

Hypertension is a common chronic medical condition; it is regarded as the most important risk factor in the global burden of disease.^1^ Globally, 1.13 million adults had hypertension in 2015, with 1 in 4 men and 1 in 5 women having hypertension.^2^ Unfortunately, the burden of hypertension continues to rise, and there is robust evidence that hypertension control substantially reduces cardiovascular morbidity and mortality.^3^,^4^ A significant proportion of patients with hypertension are diagnosed and managed in the primary care setting. For instance, most physician visits for hypertension in the United States occur in primary care;^5^ hypertension is the commonest medical disorder treated by primary care physicians in Canada^6^

The situation is similar in Nigeria; hypertension constituted 9.2% of primary care patients seen in a study in Enugu, Southeastern Nigeria.^7^ Interestingly, low- and middle-income countries have the poorest hypertension control rates (below 5%); this has been attributed to insufficient treatment and poor access to care.^8^,^9^

Furthermore, the chronic nature of hypertension makes long term follow-up a standard of care. Therefore, non-adherence to outpatient follow-up appointments reduces access to valuable care in this long continuum of care, resulting in adverse outcomes such as poor control, high risk of hospitalization, decreased clinic efficiency, and increased morbidity and mortality.^10^–^15^ A recent systematic review described the patient-, disease-, medication-, and health provider factors associated with missed follow-up appointments.^16^ However, only a few studies were conducted in the Nigerian population, and these were mainly among medical in- and outpatient specialist settings.^17^–^20^ This study aimed to assess the prevalence and predictors of non-adherence to clinic appointments among patients with poorly controlled hypertension receiving care at a family practice setting in Nigeria.

## Methods

### Study design and setting

This was a descriptive cross-sectional study. It was conducted at the Family Medicine Clinic (FMC) of a Teaching Hospital in Northwest Nigeria between June and August 2019. Nigeria operates a three-tier health care system, namely, primary, secondary, and tertiary care levels.^21^ The family physicians function at the primary and secondary care levels of the healthcare system. With a population of over 9 million people,^22^ the hospital's host state has several public (state- and federal governmentowned), private, and faith-based health care facilities. The study site (FMC) is the primary care unit of one of the federal government-owned hospitals in the state. First contact, continuous, comprehensive, and holistic health care are provided by consultants, residents, and general duty doctors of the hospital's Family Medicine Department using treatment protocols. The clinic does not operate an electronic health record nor appointment systems, but patients are seen on a “first-come, first-serve” basis. From clinic records, an average of 375 adult patients with hypertension is seen weekly at the clinic.

### Inclusion and exclusion criteria

All patients aged ≥ 18 years with a diagnosis of hypertension, who had been on treatment for ≥1 year and had a current blood pressure of ≥140/90 mmHg were included in the study. Patients with secondary hypertension, hypertensive emergencies, and pregnant women, or those who declined consent to participate were excluded.

### Sample size estimation

Using a missed-appointment prevalence of 16.7% from a study in Ibadan, Southwest Nigeria,^17^ and the formula (N=Za^2^PQ/D^2^) for estimating sample size (for observation studies with sample population ≥10,000),^23^ where N=minimum sample size, Zα=standard normal deviation corresponding to a 5% level of significance (1.96), P=0.167 and Q=1-P and D= level of precision, set at 5%, a minimum sample size of 214 was obtained. With an estimated clinic population of patients with hypertension of 4500 in the 12-weeks study period, the formula for calculating sample size for studies with a sample population < 10,000 [N/(1+N/n)] was used (n=sampling frame, 4500).^23^ A minimum sample size of 226 was obtained after 10% was added to increase the study power.

### Sampling technique and study procedure

On each clinic day, a systematic sampling technique was employed to select every 20^th^ patient (sampling frame/sample size, 4500/226 ≈20) who met the inclusion criteria from a list of patients with hypertension identified at triage by a trained research assistant. This was done after the first participant had been selected by balloting.

Each participant received an explanation of the study objectives; written informed consent was obtained before the investigator administered the study questionnaire. The participants' reason for the current clinic visit was also managed, irrespective of their participation in the study. An average of 20 patients was recruited weekly until the required sample size was reached.

### Data collection tool

Participant information was collected using a pretested, investigator-administered questionnaire developed following an extensive literature search. The questionnaire's internal validity was derived using Cronbach alpha, and a value of 0.80 was obtained. It explored participants' (a) sociodemographic characteristics such as age, sex, residential location, perception of distance between residential location and the clinic, mode of transport to hospital, health insurance status, who pays medical bills, current employment status, current marital status, and family type); (b) clinical characteristics (e.g., duration of hypertension treatment, presence of comorbidities, type of comorbidities, number of comorbidities, current blood pressure, number of current daily pills, medication adherence); (c) clinic-appointment characteristics (e.g., mode of communicating appointments, appointment frequency, having ever missed an appointment, average number of missed appointments out of ten, reason/s for missing appointments); (d) perception regarding physicians' attempts at explaining the disease; (e) awareness of the need to keep appointments; and (f) awareness of consequences of missed appointments.

### Definition and measurement of variables

(a) The primary outcome variable was clinic appointment non-adherence. It was defined as the tendency to miss more than three clinic appointments out of every ten clinic appointments during the patients' lifetime.^17^,^18^ This was assessed using the question “Out of every ten clinic appointments, on average, how many do you miss?”

(b) The presence of comorbidity was assessed by reviewing participants' medical records.

(c) Participants' blood pressures were measured by the principal investigator following standard protocol.^24^

(d) The shortest distance between participants' home and the clinic was assessed using the global positioning system (GPS).^25^

(e) Awareness of the consequences of missed clinic-appointment was assessed using nine closed-ended questions (Appendix),^17^ (with responses of Yes, No, or I don't know). A correct answer received a score of one, while a wrong or no response received a score of zero; a total score of ≥7 out of a maximum of 9 was defined as “sufficient awareness,” whereas a score of < 7 was “insufficient awareness”.

### Ethical considerations

Ethical approval was obtained from the Research Ethics Committee of Aminu Kano Teaching Hospital (No.: NHREC/21/08/2008/AKTH/EC/2435). In addition, written informed consent was obtained from each participant. Participation was voluntary, and participants' information was kept confidential.

### Data analysis

Data were entered and analyzed using Epi Info Version 7.1.1.14 (2013; CDC, Atlanta, GA). Continuous variables were summarized using means and standard deviations. Categorical variables were presented in frequency tables. A Chi-square test was used to determine the association between categorical variables and clinic appointment non-adherence. Multivariate logistic regression was done to determine the predictors of clinic appointment non-adherence. P-value was set at less than 0.05.

## Results

### Sociodemographic characteristics of the participants

A total of 234 eligible patients were recruited during the study period and were analyzed. The participants' mean age was 55±12.2 years (range: 23–85 years). Most participants were females (163, 69.7%), and they lived >5 kilometres from the hospital (158, 67.5%); but, 125 (53.4%) participants felt they lived far from the hospital (Table 1). Their commonest mode of transport to hospital was commercial vehicles (166, 70.9%); more participants had no formal education (90, 38.5%); while half were unemployed. Most participants (211, 90.2%) had no health insurance, while only 90 (38.5%) participants paid the medical bills by themselves.

**Table 1:** Sociodemographic characteristics of participants (n=234)

Variable	n (%)
**Age (years) [mean = 55±12.2]**	
**20–29**	3 (1.3)
**30–39**	23 (9.8)
**40–49**	49 (20.9)
**50–59**	65 (27.8)
**≥ 60**	94 (40.2)
**Sex**	
**Male**	71 (30.3)
**Female**	163 (69.7)
**Residential location from hospital** **(Km)**	
**≤ 5**	76 (32.5)
**>5**	158 (67.5)
**Perceived home distance from hospital**	
**Near**	108 (46.2)
**Far**	125 (53.4)
**Not sure**	1 (0.4)
**Usual mode of transport to hospital**	
**Private**	68 (29.1)
**Commercial**	166 (70.9)
**Educational level**	
**None**	90 (38.5)
**Primary**	46 (19.6)
**Secondary**	56 (23.9)
**Tertiary**	42 (18.0)
**Employment status**	
**Employed** *	105 (44.9)
**Retired civil servant**	12 (5.1)
**Unemployed**	117 (50.0)
**Marital status**	
**Single**	4 (1.7)
**Married**	161 (68.8)
**Divorced/separated**	7 (3.0)
**Widow**	62 (26.5)
**Family type**	
**Monogamous**	120 (51.3)
**Polygamous**	110 (47.0)
**Not applicable**	4 (1.7)
**Do you have health insurance?**	
**No**	211 (90.2)
**Yes**	23 (9.8)
**Who pays your medical bills?**	
**Children**	84 (35.9)
**Other relations**	60 (25.6)
**Myself**	90 (38.5)

* self-employed, working for private or government organizations.

### Clinical characteristics of the participants

Most participants (103, 44.0%) had received treatment for hypertension for 1–5 years (Table 2), 102 (43.6%) participants had comorbidity.

**Table 2:** Clinical characteristics of participants (n=234)

Variable	n (%)
**Treatment duration (years) Mean** **8.8±7.6**	
**1–5**	103 (44.0)
**6–10**	72 (30.8)
**>10**	59 (25.2)
**Presence of comorbidity**	
**No**	132 (56.4)
**Yes**	102 (43.6)
**Number of chronic diseases**	
**1 (hypertension only)**	132 (56.4)
**2**	91 (38.9)
**3**	10 (4.3)
**4**	1 (0.4)
**Types of comorbid conditions** *	
**Diabetes**	44 (18.8)
**PUD**	24 (10.3)
**Joint pain**	22 (9.4)
**HHD/CCF**	9 (3.9)
**Others** **	16 (6.9)
**Current blood pressure (mmHg)**	
**SBP (mean: 160.7±16.1)**	-
**<160**	96 (41.0)
**.160**	138 (59.0)
**DBP (mean: 95.8±11.0)**	
**<100**	105 (48.9)
**≥100**	129 (55.1)
**Current number of daily pills**	
**Mean 2.8±1.1**	-
**1–3**	182 (77.8)
**4–7**	52 (22.2)

* Some respondents had > 1 morbidity

** Asthma (4), benign prostatic hypertrophy (3), cataract (2), stroke (2), depression (2), dyslipidemia (2), obesity (1), seizure disorder (1). PUD: peptic ulcer disease; HHD: hypertensive heart disease; CCF: congestive cardiac failure; SBP: systolic blood pressure; DBP: diastolic blood pressure.

Type 2 diabetes mellitus was the commonest comorbidity (44, 18.8%). Their mean systolic blood pressure (SBP) and diastolic blood pressure (DBP) was 160.7±16.1 mmHg and 95.8±11.0 mmHg, respectively; a majority had a SBP of ≥ 160mmHg (138, 69.0%) and a DBP of ≥100mmHg (129, 55.1%). Most participants (182, 77.8%) were on 1–3 pills daily.

### Clinic appointment characteristics of the participants

Most participants received treatment from the study site alone (155, 66.2%) (Table 3). Appointments were communicated verbally to most participants (162, 69.2%). Most of the participants had previously missed a clinic appointment (157, 67.1%); however, 60 (25.6%) participants missed > 3 out of every 10 appointments, whereas 174 (74.4%) participants were adherent to their appointments. Reasons reported for missed appointment were mainly lack of funds for transportation/drugs (26.9%), lack of symptoms (16.7%), conflict with work schedule (9.8%), distance of hospital from home (8.6%), and travelled out of town (6.9%). Most participants reported receiving explanations on their diagnosis (hypertension) (175, 75.1%), complications of hypertension (154, 65.8%), and treatment options of hypertension (143, 61.1%). Additionally, most participants (191, 81.6%) were aware of the need for follow-up visits, but only 128 (54.7%) were sufficiently aware of the consequences of missed appointments.

**Table 3:** Clinic appointment characteristics of participants (n=234)

Variable	n (%)
**No. of clinics usually visited**	
**1**	155 (66.2)
**2–4**	79 (33.8)
**Usual mode of communicating appointments**	
**Written**	9 (3.9)
**Verbal**	162 (69.2)
**Both**	63 (26.9)
**Interval of appointments**	
**2-weeks**	48 (20.5)
**1-month**	121 (51.7)
**2-months**	44 (18.8)
**3-months**	11 (4.7)
**Wasn't told**	10 (4.3)
**Ever missed a clinic appointment?**	
**No**	77(32.9)
**Yes**	157 (67.1)
**No. of missed appointments in 10**	
**None (adherent)**	77 (32.9)
**1–3 (adherent)**	97 (41.5)
**>3 (nonadherent)**	60 (25.6)
**Reasons for missed appointments** *	
**Lack of money for transport/drugs**	63 (26.9)
**Lack of symptoms**	39 (16.7)
**Conflict with work schedule**	23 (9.8)
**Distance**	20 (8.6)
**Travelled**	16 (6.9)
**Delays in hospital**	9 (3.9)
**Forgetfulness**	8 (3.4)
**Nobody to bring me**	8 (3.4)
**Visits another hospital**	7 (3.0)
**Fear of hospital**	5 (2.3)
**Others** **	7 (3.0)
**Received explanation of hypertension** **diagnosis**	
**No**	58 (24.9)
**Yes**	175 (75.1)
**Received explanation on hypertension** **complication**	
**No**	80 (34.2)
**Yes**	154 (65.8)
**Received explanation on treatment options**	
**No**	91 (38.9)
**Yes**	143 (61.1)
**Awareness of the need for follow up visits**	
**No**	43 (18.4)
**Yes**	191 (81.6)
**Awareness of consequences of missed** **appointments**	
**Insufficient**	106 (45.3)
**Sufficient**	128 (54.7)

* Reasons were either alone or in combinations.

** Yet to finish medicines (3); Public holiday, refilled drugs by myself, health workers' strike and requested laboratory results not ready (1 each)

### Factors associated with clinic appointment non-adherence

Table 4 shows that participants' employment status (χ^2^=7.47, *P*=0.006), who pays medical bills (χ^2^=5.15, *P*=0.023), and SBP (χ^2^=8.57, *P*=0.003) had a statistically significant association with clinic appointment non-adherence.

**Table 4:** Factors associated with clinic appointments non-adherence among participants (n=234)

Variable	Clinic Appointment		χ^2^	p- value
	Non- adherence n (%)	Adherence n (%)		
**Age (years)**				
**<60**	41 (68.3)	99 (56.9)	2.43	0.119
**≥ 60**	19 (31.7)	75 (43.1)		
**Sex**				
**Male**	20 (33.3)	51 (29.3)	0.34	0.559
**Female**	40 (66.7)	123 (70.7)		
**Perceived home** **distance from** **hospital**				
**Near**	27 (45.0)	81 (46.5)	FET	0.816
**Far**	33 (55.0)	92 (52.9)		
**Not sure**	0 (0.0)	1 (0.6)		
**Residential location** **from hospital** **(Km)**				
**≤ 5**	21(35.0)	55 (31.6)	0.23	0.629
**>5**	39 (65.0)	119 (68.4)		
**Educational level**				
**Low (None, Primary)**	33 (55.0)	103 (59.2)	0.32	0.570
**Higher (Secondary,** **Tertiary)**	27 (45.0)	71 (40.8)		
**Employment status**				
**Employed**	36 (60.0)	69 (39.7)	7.47	0.006*
**Unemployed** **(unemployed/retirees)**	24 (40.0)	105 (60.3)		
**Marital status**				
**Single**	1 (1.7)	3 (1.7)	FET	0.478
**Married**	46 (76.6)	115 (66.1)		
**Divorced/separated**	1 (1.7)	6 (3.5)		
**Widow**	12 (20.0)	50 (28.7)		
**Family type**				
**Monogamous**	27 (45.0)	93 (53.4)	FET	0.213
**Polygamous**	33 (55.0)	77 (44.3)		
**Not applicable**	0 (0.0)	4 (2.3)		
**Usual mode of** **transport to hospital**				
**Private**	14 (23.3)	54 (31.0)	1.29	0.257
**Commercial**	46 (76.7)	120 (69.0)		
**Do you have** **health insurance?**				
**No**	56 (93.3)	155 (89.1)	FET	0.340
**Yes**	4 (6.7)	19 (10.9)		
**Who pays your** **medical bills?**				
**Other relations**	22 (36.7)	38 (21.8)	5.15	0.023*
**Self/Children**	38 (63.3)	136 (78.2)		
**Duration of hypertension** **treatment** **(years)**				
**1–10**	47 (78.3)	128 (73.6)	0.54	0.463
**>10**	13 (21.70)	46 (26.4)		
**Presence of** **comorbidity**				
**No**	36 (60.0)	96 (55.2)	0.42	0.516
**Yes**	24 (40.0)	78 (44.8)		
**Current blood** **pressure (mmHg)**				
**SBP**				
**<160**	15 (25.0)	81 (46.6)	8.57	0.003*
**≥160**	45 (75.0)	93 (53.4)		
**DBP**				
**<100**	26 (43.3)	79 (45.4)	0.08	0.781
**≥100**	34 (56.7)	95 (54.6)		
**Current number** **of daily pills**				
**≤3**	44 (73.3)	138 (79.3)	0.92	0.337
**>3**	16 (26.7)	36 (20.7)		
**No. of hospitals** **usually visited**				
**1**	41(68.3)	114 (65.5)	0.16	0.691
**≥2**	19 (31.7)	60 (34.5)		
**Mode of communicating** **appointment**				
**Written**	2 (3.3)	7 (4.0)	FET	0.530
**Verbal**	45 (75.0)	117 (67.2)		
**Both**	13 (21.7)	50 (28.8)		
**Interval of last** **appointment**				
**2-weeks – 1** **month**	41 (68.3)	128 (73.6)	3.28 df=2	0.194
**2–3 months**	14 (23.3)	41 (23.6)		
**Wasn't told**	5 (8.3)	5 (2.9)		
**Received explanation** **of hypertension** **diagnosis**				
**No**	15 (25.4)	43 (24.7)	0.12	0.913
**Yes**	44 (74.6)	131 (75.3)		
**Received explanation** **on hypertension** **complications**				
**No**	23 (38.3)	57 (28.4)	0.62	0.432
**Yes**	37 (61.7)	117 (67.2)		
**Received explanation** **on treatment** **options**				
**No**	24 (40.0)	67 (38.5)	0.04	0.838
**Yes**	36 (60.0)	107(61.5)		
**Awareness of the** **need for follow up** **visits**				
**No**	15 (25.0)	28 (16.1)	2.36	0.124
**Yes**	45 (75.0)	145 (83.9)		
**Awareness of** **consequences of** **missed appointments**				
**Insufficient**	30 (50.0)	76 (43.7)	0.72	0.396
**Sufficient**	30 (50.0)	98 (56.3)		

χ^2^: Chi square test; FET: Fisher's exact test

* significant

df: degree of freedom. SBP: systolic blood pressure; DBP: diastolic blood pressure.

### Predictors of non-adherence to clinic appointments

The multivariate logistic regression analysis of variables that had statistically significant associations with non-adherence to clinic appointments is shown in Table 5. Participants who had employment were two times more likely to be non-adherent to clinic appointments compared to those without employment (OR [odds ratio] =2.93, 95% CI [confidence interval] = 1.53–5.63 *P*=0.001). Similarly, participants whose medical bills were paid by other relations (excluding the participants and their children) were two times more likely to be non-adherent to clinic appointments (OR=2.86, 95%CI=1.41–5.80, *P*=0.004). However, participants with a SBP of <160 mmHg were less likely to be non-adherent to clinic appointments (OR=0.36, 95%CI=0.18–0.70, *P*=0.003); this also suggest that participants with a SBP ≥ 160mmHg were more likely to be non-adherent to clinic appointments.

**Table 5:** Predictors of non-adherence with clinic appointments (n=234)

Variable	OR	95% CI	Coefficient	p-value
**Employment status**				
**(Employed/Unemployed)**	2.93	1.53–5.63	1.08	0.001*
**Who pays medical bills?**				
**[Other relations/** **(Self/children)/]**	2.86	1.41–5.80	1.05	0.004*
**Systolic blood pressure** **(mmHg)**				
**<160 / ≥160**	0.36	0.18–0.70	-1.03	0.003*
**Constant**	-	-	0.43	0.306

OR: odds ratio; CI: confidence interval

* significant

## Discussion

This study examined the prevalence and predictors of non-adherence to clinic appointments among patients with poorly controlled hypertension in a Nigerian primary care setting. It found a clinic appointment non-adherence rate of 25.6%. Factors such as being employed, patient's or their children's inability to pay medical bills, and current systolic blood pressure of ≥160 mmHg were the predictors of non-adherence to clinic appointments.

About a quarter of our study participants (25.6%) were non-adherent to clinic appointments. This finding was less than the prevalence of 31% found in a retrospective 12-month clinic attendance chart review of hypertensive patients attending a community health centre. However, it was higher than the 20% reported among medical inpatients with severe hypertension in the US,^18^,^19^ and the 16.7% reported in hypertensive medical outpatients in Ibadan, Southwestern Nigeria.^17^ This suggests that non-adherence to clinic appointments remains a significant challenge encountered by physicians providing care to patients with hypertension in medical outpatient clinics, medical in-patient settings, and primary care/family practice settings. The difference between our study prevalence and those found in the other studies could be due to differences in study population and design.

Furthermore, being employed in this study was a predictor of non-adherence to clinic appointments. This finding was remarkable because recent studies (literature <15 years) have not found a significant association between employment status and non-adherence to clinic appointments among patients with hypertension.^16^–^18^ We are unsure if our study population of only uncontrolled hypertension is responsible for this association. Again, while the link between having employment and appointment non-adherence is unclear, we suspect that the conflict of clinic appointments with work-schedule reported as a reason for missed clinic appointments by some participants in our study, and some other studies could partly explain this finding.^16^ This conflict could be due to the absence of a flexible appointment system that allows employees to access health care at their convenience in our public primary care clinics.

In addition, participants whose medical bills could not be paid by themselves, or their children were two times more likely to be non-adherent to clinic appointments. This finding shows the important role financial difficulties and lack of family support can pose to the management of chronic medical conditions such as hypertension.^18^,^26^

Besides providing funds to pay for the treatment of hypertension, the absence of social support provided by close family members (e.g., their children) could be responsible for this effect on non-adherence to appointments. These close family members can remind them of their appointments and sometimes convey them to the clinic; some participants cited “forgetfulness” and “nobody to take me to the hospital” as reasons for missing their appointment. Although participants' social support was not measured in this study, its absence could partly explain the statistically insignificant association observed between health insurance status (which provides payment for medical bills) and appointment non-adherence in this study compared to other studies where having health insurance reduced appointment non-adherence.^18^

Furthermore, participants with a systolic blood pressure of more than 160mmHg were more likely to be non-adherent to clinic appointments. This finding was similar to the result obtained in the study that assessed ethnic differences in appointment-keeping in the Diabetes Study of Northern California, where systolic blood pressure greater than 130 mmHg was associated with poor appointment keeping.^27^ It is also similar to the result of the study among in-patients of African-American descent, in which a higher mean diastolic blood pressure of 127.1±14.1 mmHg was associated with non-adherence to clinic appointments.^18^ Nonetheless, this finding has an uncertain role in predicting patients' non-adherence to clinic appointments because of the multiple intertwined factors associated with clinic appointment non-adherence. These factors may include being employed, lack of health insurance, inadequate knowledge, attitude and belief about hypertension, poor medication adherence and blood pressure control, and seeking care elsewhere because of the development of complications.^16^

Furthermore, this study found that patient variables such as age, sex, educational level, marital status, family type, the distance between home and clinic, treatment duration, comorbidity, mode of communicating appointment, appointment intervals, receipt of explanation on hypertension, awareness of the need for follow-up visits and consequences of missed appointments had no statistically significant association with non-adherence to clinic appointments contrary to previous systematic review.^16^ This could be due to differences in study populations and designs.

### Recommendations

With a high appointment non-adherence rate of 25.6%, interventions such as patient education, short message service/ text messaging, and mobile phone application reminders should be considered in improving appointment adherence in line with current evidence in developed countries.^28^ This may be feasible because of the high penetration of mobile telephones in Nigeria.^29^,^30^ However, the use of these digital interventions will require local randomized controlled trials to ascertain their effectiveness. Patients with hypertension with increased risk for non-adherence to clinic appointments such as those with employment, those whose medical bills are not paid by themselves or their children, and those with higher systolic blood pressures [≥160 mmHg]) can form the target population for these digital interventions.^31^ Furthermore, with the high proportion of patients with hypertension with employment (44.9%) in this clinic, a flexible appointment system should be considered; however, its effectiveness requires further investigation.

Finally, family physicians may need to improve the deployment of the social support systems of patients with hypertension. Evidence supports the involvement of close family members of hypertensive patients, such as their children, spouses, and significant others, in optimizing the control of hypertension.^26^

### Study limitations

This study had some limitations. Firstly, it was carried out in an urban facility; hence findings may differ in primary care clinics in rural settings. Secondly, as in other studies, a lifetime missed clinic appointment was self-reported; thus, clinic appointment adherence could have been overestimated in some cases.

## Conclusion

The appointment non-adherence rate was high. Being employed, patients' or their children's inability to pay medical bills, and systolic blood pressure of >160 mmHg were predictors of clinic appointment non-adherence. Identifying these predictors among patients with poorly controlled hypertension in the primary care clinic and providing effective interventions that address them can be important ways of reducing clinic appointment non-adherence.
